# Virtual monitoring in CF – the importance of continuous monitoring in a multi-organ chronic condition

**DOI:** 10.3389/fdgth.2023.1196442

**Published:** 2023-05-04

**Authors:** Tamara Vagg, Kevin F. Deasy, Wendy W. Chapman, Sarath C. Ranganathan, Barry J. Plant, Shivanthan Shanthikumar

**Affiliations:** ^1^ Cork Centre for Cystic Fibrosis (3CF), Cork University Hospital, Cork, Ireland; ^2^ HRB Clinical Research Facility Cork, University College Cork, Cork, Ireland; ^3^ Department of Medicine, University College Cork, Cork, Ireland; ^4^ The Centre for Digital Transformation of Health, University of Melbourne, Melbourne, VIC, Australia; ^5^ Respiratory and Sleep Medicine Department, Royal Children’s Hospital, Melbourne, VIC, Australia; ^6^ Respiratory Diseases Research, Murdoch Children’s Research Institute, Melbourne, VIC, Australia; ^7^ Department of Paediatrics, The University of Melbourne, Melbourne, VIC, Australia

**Keywords:** cystic fibrosis, virtual monitoring, chronic condition, remote monitoring, review

## Abstract

Cystic Fibrosis (CF) is a chronic life-limiting condition that affects multiple organs within the body. Patients must adhere to strict medication regimens, physiotherapy, diet, and attend regular clinic appointments to manage their condition effectively. This necessary but burdensome requirement has prompted investigations into how different digital health technologies can enhance current care by providing the opportunity to virtually monitor patients. This review explores how virtual monitoring has been harnessed for assessment or performance of physiotherapy/exercise, diet/nutrition, symptom monitoring, medication adherence, and wellbeing/mental-health in people with CF. This review will also briefly discuss the potential future of CF virtual monitoring and some common barriers to its current adoption and implementation within CF. Due to the multifaceted nature of CF, it is anticipated that this review will be relevant to not only the CF community, but also those investigating and developing digital health solutions for the management of other chronic diseases.

## 1. Introduction

**Figure 1:**
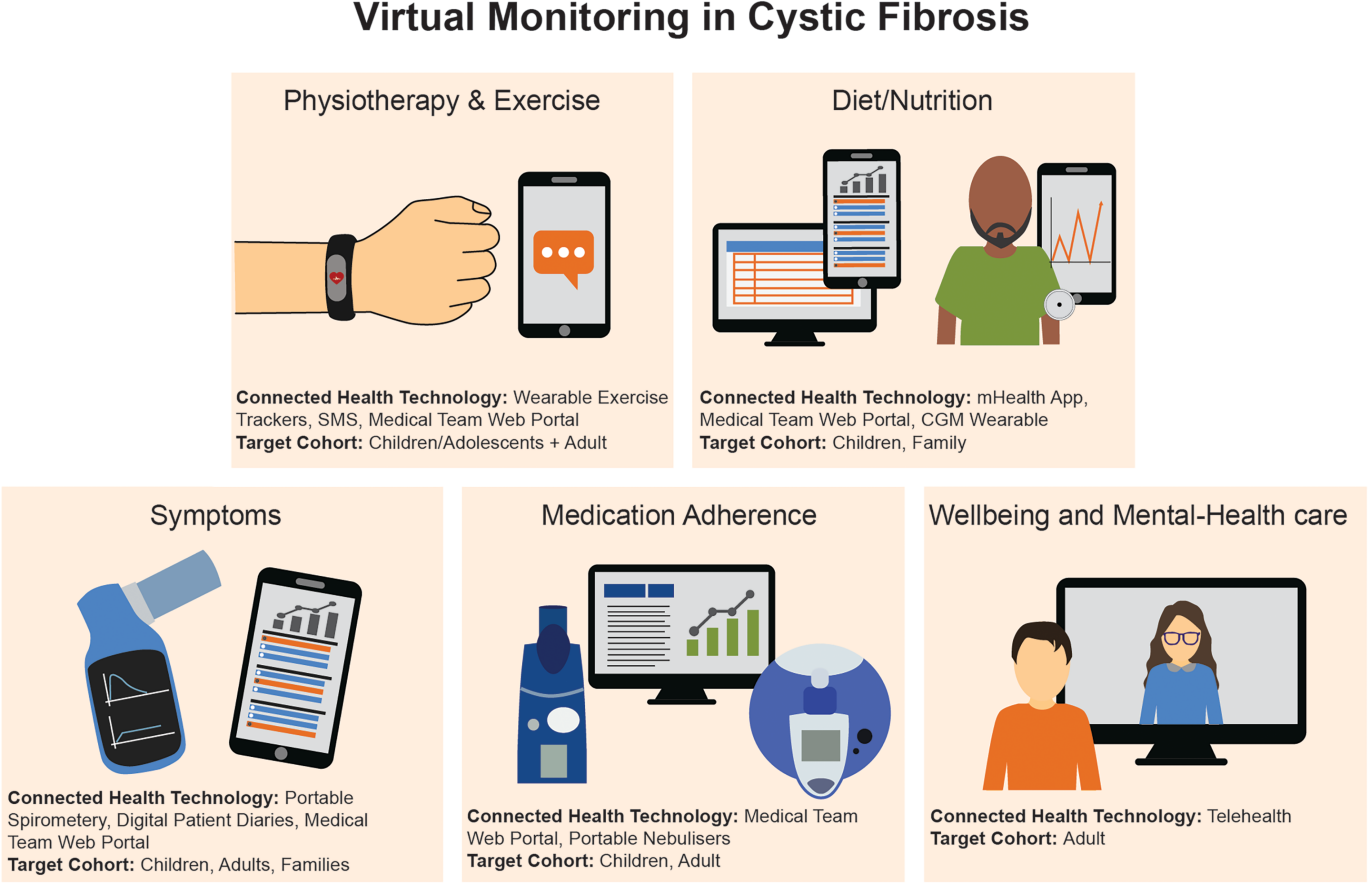
Summary of the virtual monitoring devices and target cohorts within CF.

Cystic Fibrosis (CF) is a chronic, autosomal recessive, life-limiting multisystem disease. In the lung, CF causes abnormal mucus clearance resulting in increased infection and inflammation. In turn, this results in irreversible lung damage which is the primary reason for the reduced life expectancy experienced by People with CF (PwCF). Other manifestations of CF include exocrine pancreatic insufficiency (resulting in the need for pancreatic enzyme supplementation), CF-related diabetes (most commonly treated with insulin), and psychological conditions. As such patients must adhere to strict medication regimens, physiotherapy, and diet on a daily basis, with one study finding the average adult with CF spends two hours per day on CF care (1). In addition, PwCF attend regular clinic appointments to manage their condition. In between clinic appointments, PwCF must manage their burden of care themselves, and further, must self-monitor for pulmonary exacerbations which need additional treatment and can result in accelerated lung damage. Digital health technologies offer the ability to assist PwCF with their current care burden, as well as more proactively monitor their health status in between clinic visits. This review highlights key virtual monitoring studies (summarised in Table 1 and Figure 1) within the area of CF.

**Table 1:** A summary of the studies outlined in this review for CF virtual monitoring.

CF virtual monitoring focus	No. of studies	Evaluation methods	Positive results	Negative results/Limitations
Physiotherapy & Exercise	5	Feasibility, Acceptability	Physical activity trackers are acceptable for both paediatric patients and medical professionals. Accompanying encouraging messages from medical teams can improve activity.	While the messages can improve activity, there was no significant effect on clinical parameters
Diet & Nutrition	2	Clinical Parameters	MyCyFAPP – has generated educational resources to support families and patients with nutrition. It has also validated a model for individual PERT calculations and shown to improve quality of child's diet. CGM – improved glycaemic control compared to finger prick glucose monitoring	Limited data regarding long-term effectiveness of intervention. No change in body mass index. Diet still does not meet criteria for healthy or adequate diet. Studies showed a high risk of bias, including publication bias, and selective reporting of results. No data yet to support benefits on pulmonary status or quality of life.
Monitoring Symptoms	5	Feasibility, Usability, Acceptability, Clinical Parameters	It has been found to be feasible, acceptable, and usable. PEx can be detected sooner. It can potentially prevent the need for IV antibiotics.	While PEx could be detected sooner, it did not prevent lung function decline. Sustained use and even adoption of virtual monitoring is a challenge
Medication Adherence	2	Feasibility, Clinical parameters	It has the potential to improve medication adherence.	Patient adherence to manual data entry can be low and automatic data transfer is required.
Wellbeing & Mental-Health	2	Pilot study of clinical parameters, Feasibility, Acceptability	Remote mental health therapy can be as effective as in person therapy. Virtual CBT is acceptable and feasible	Publicly available mHealth apps to support this focus are found to be low quality.

PERT, pancreatic enzyme replacement treatment; CGM, continuous glucose monitoring; PEx, pulmonary exacerbations; IV, intravenous; CBT, cognitive behavioural therapy.

Virtual monitoring, like many terms within the digital health space, can have multiple meanings or be used interchangeably with other terms such as remote monitoring and continuous monitoring. For the purpose of this review, virtual monitoring refers to remote collection of medical and health data that can be used to guide patient care in a pre-emptive or proactive manner (2). PubMed, Web of Science and Scopus were the electronic databases utilized and the search terms used to identify the below manuscript are included in Box 1. There was no restriction on the year for publication.

Box 1 **Search Terms used to identify the enclosed manuscripts.**

“Cystic Fibrosis” AND (“Virtual Health” OR “Remote Monitoring” “TeleMonitoring” OR “Digital Health” OR “Home Monitoring” OR “ehealth” OR “telehealth” OR “telemedicine” OR “wearable devices” OR “Virtual Care”).

## 2. Virtual monitoring in CF care

### 2.1. Virtual monitoring for CF physiotherapy & exercise

PwCF are recommended to do physiotherapy and aerobic exercise daily (3). Virtual monitoring can support patients to complete these tasks. In 2018 Shelley et al*.* surveyed children/adolescents with CF and their clinicians in the UK and found that physical activity monitoring devices were acceptable to both patients and medical professionals and can contribute to better understanding of the barriers preventing exercise (4). In Ireland in 2022 Curran et al*.* provided adult CF patients with a Fitbit Charge 2 (Fitbit Charge 2; Fitbit, California, US) and set goals for the number of steps/activity to achieve in a week. The participants were then randomly assigned into two groups, the first had their data monitored by the CF team and were sent weekly encouraging text messages, the second received the activity tracker only. Interestingly, there was a significant increase in step count in the group who received the text messages, but there was no significant effect on clinical parameters (5). There are currently three large multicentre studies investigating different physical activity monitoring with CF children including portable devices (project Fizzyo) (6), telehealth (project CyFit telehealth) (7), and web-based activity trackers (project ActionPACT) (8), but findings are yet to be published.

### 2.2. Virtual monitoring for CF diet/nutrition

A CF specific diet is a key aspect of CF care, and one of the strongest exemplars of virtual monitoring to support CF nutrition self-management is the MyCyFAPP, an international collaboration between 12 European organisations (9, 10). This system encompasses a patient focused mobile health (mHealth) application to disseminate educational and nutrition information for parents of children with CF, educational games for children with CF, diet trackers, and Pancreatic Enzyme Replacement Treatment (PERT) calculators. This mHealth system also has an integrated web-tool to display variable analytics to medical professionals, while simultaneously allowing CF dietitians to send recommendations/corrections to the patient. The latest iteration of this research includes a predictive model for optimal PERT adjustment to provide patients with their own specific number of enzymes per meal/food product. To date, use of the MyCyFAPP has shown benefit. Children had their diet analysed before using the app, and six months after using the app. The pre and post data comparison found that the app led to better macronutrient distribution (more protein and fat, less carbohydrates) and less intake of ultra-processed food. Another common example of virtual monitoring in CF dietetics is the use of Continuous Glucose Monitoring (CGM) devices in those with CF related diabetes. These devices are worn by the patient and continuously read glucose levels and display these data in a report found on an accompanying app. A recent systematic review found that CGM may improve glycaemic control in CF related diabetes compared to standard finger prick glucose monitoring (11).

### 2.3. Virtual monitoring of CF symptoms

Symptom monitoring using portable devices (e.g., a spirometer) and/or Patient Reported Outcome (PRO) tools for the early detection of Pulmonary Exacerbations (PEx), is one of the most investigated areas within CF virtual monitoring. Early detection and management of PEx is crucial to preventing lung damage. One of the earliest examples of this began in 1984 through to 1992 where patients recorded symptoms on paper at home, which were later sent to a data centre and transcribed digitally. This early study identified that monitoring patients in this way did not cause any negative impact on the patient and could slow down lung function decline when compared to a control group whose symptoms were not monitored in this way (12).

Later in 2010 with the emergence of more sophisticated technology, researchers in the UK furthered this concept with a digital diary that allowed for the manual insertion of spirometry and symptom values that could then be sent in real time to the medical team for monitoring potential PEx. In this study, adult patients were asked to monitor and enter these data daily, resulting in poor uptake (37%) and the potential introduction of human error. However, for those who completed the study, PEx were detected earlier and treated via oral antibiotics which may have prevented the need for intravenous antibiotics (13).

In 2013, a web-based symptom monitoring diary was developed by a multi-disciplinary team from Tasmania, “myCF”. This allowed symptom data to be collected and shared with health professionals and health mentors. Unlike other symptom monitoring approaches to date, myCF also incorporated educational materials, was linked to a patient forum, and targeted both paediatric and adult patients, along with their families. An initial pilot study of the system found that myCF showed promise in complimenting standard care by facilitating the development of self-care skills for patients and families with geographical restrictions to care, and while it could help with peer support the idea of virtual human connection required further review (14).

In 2017, smartphone technology, or “mHealth”, was introduced into symptom monitoring for CF. This was achieved via an app that contained a symptom questionnaire which could send the data to the healthcare team for analysis and identification of PEx. Again, only the usability of this system was evaluated with a small adult cohort (*n* = 10) with positive usability results for the use of this mHealth app for symptom monitoring (15). In the same year the results of the eICE study were also published. This multicentre randomised trial recruited 267 adolescent/adult PwCF, participants were randomised to standard care or an intervention which consisted of regular patient reported symptoms and home spirometric data which was collected via a modified spirometer (capable of recording and sending lung function and symptom data). More PEx were detected in the intervention arm, but there was no difference between groups in lung function decline (16).

More recently in 2022 the CLIMB-CF study also investigated symptom monitoring via a smartphone app in children, adolescents, and families during the COVID-19 pandemic. This app recorded symptoms via questionnaires and connected Bluetooth® equipment (activity tracker, weighing scales, pulse oximeter, thermometer, spirometer). This multicentre study recruited 144 PwCF/families to evaluate feasibility, acceptance, and mental health impact. While there was no negative impact on the lives of the patients/families, the adoption of the app was variable and by month two of the six month study, study adherence had dropped from >50% to 20%, demonstrating that it was not sustained over time (17).

### 2.4. Virtual monitoring of CF medication adherence

Adherence in CF can be challenging due to the complexity of the treatment regimens, the frequency of treatment, and the potential side effects of medications. Factors that can influence adherence include patient motivation, social support, healthcare provider communication, and access to healthcare resources.

Digital technology can be a powerful tool for adherence monitoring in CF (18). The use of smart nebuliser devices is the most studied technology for monitoring adherence in CF. The eTrack rapid nebuliser (eTrack; Pari Pharma GmBH, Starnberg, Germany) is a portable device used for CF care as part of the CFHealthHub project. It uses a nebuliser with a built-in sensor to record nebuliser compliance and then sends the data automatically to a web-based digital platform to be viewed by both the patient and clinical team (19). The design of the CFHealthHub was informed by qualitative interviews with PwCF who used the system during a pilot study. It was then later rolled out to 19 CF centres (20).

The importance of making the process of self-monitoring entirely automated is highlighted by Thornton et al. who evaluated the I-neb nebuliser (I-neb; Phillips, Amsterdam, Netherlands). This smart nebuliser does not perform automatic data transfer, which was found to be a barrier to using the device to improve treatment adherence as >50% of patients failed to upload data to the online portal regularly (21).

### 2.5. Virtual monitoring for CF wellbeing and mental-health care

Like most chronic conditions, PwCF and their families are at greater risk of mental health issues due to multiple factors (22). Additionally, there are significant associations between positive mental health and self-reported physical health and quality of life (23). As such, much research investigating virtual monitoring for overall CF self-management often report the effects on overall depression and anxiety levels in patients. However, specific digital health interventions for CF mental health are lacking (24, 25). Amerio et al. in 2020 further suggested that with the recent advances in CF therapies, CF care is at a pivotal time to move from secondary prevention of mental health issues, and instead move to predicting patients' susceptibility and pro-actively treating (26). The concept of integrating predictive models could lend itself well to virtual monitoring techniques, however to date such a digital health system is yet to be created for CF care. Instead, telehealth technologies are currently being used to deliver remote counselling without monitoring or recording of additional data. One example of this is the Acceptance and Commitment Therapy (ACT) telehealth study launched in 2021 which delivered six virtual or in person sessions to adult CF patients. The initial pilot study found that the virtual sessions were as effective as in-person sessions, and improved both psychosocial functions and lung function post-treatment (24). Similarly in 2021 and 2022, online Cognitive Behavioural Therapy for CF (CF-CBT) was delivered to adult CF patients. Pilot data from these studies also demonstrate that the online CF-CBT program was both feasible and acceptable (27). In addition to the sparsity of available research for this CF focus, a systematic review in 2021 investigating mHealth apps for CF emotional and physical health concluded that current available research was of low quality due to small sample sizes, limited to pilot studies, and with homogeneity in data (28).

## 3. Discussion

### 3.1. Future directions for CF virtual monitoring

Many of the virtual monitoring examples encompassed in this review pre-date the COVID-19 pandemic and more importantly, the advent of widespread availability of highly effective modulator therapy. Modulator therapies are drugs which specifically target the defective ion channel which is responsible for the manifestations of CF. While such therapy does not cure the condition, patients are exhibiting improvements in key health parameters such as lung function, weight gain and decrease in pulmonary exacerbations. These revolutionary medicines have improved quality of life in patients and are allowing patients to live longer, healthier lives. Consequently, there is an increase in PwCF seeking employment, travelling abroad, attending third level education, and family planning. This pivotal time in CF care also provides a catalyst for the next generation of CF virtual monitoring to support and meet the changing needs of all CF stakeholders. As such this section will discuss potential future developments in the field of CF virtual monitoring.

#### 3.1.1. New data analysis techniques

Artificial intelligence (AI) has several potential future applications (29). Virtual monitoring has the potential to enrich data available for machine learning using not only more granular patient generated metrics (portable spirometry, blood sugar readings, etc.), but also through the use of patient generated information such as patient symptom reports, symptom diaries and notes.

One specific application of AI in virtual monitoring for CF is the use of predictive analytics. Predictive models have been studied in CF in the past (30, 31). Predictive analytics involves the use of Machine Learning (ML) algorithms to analyse patient data and predict future outcomes, such as the risk of hospitalisation or the likelihood of treatment success. This information can be used by healthcare providers to make informed decisions about the patient's treatment plan. An unvalidated proof of concept of this has already been developed using CF registry data in the UK to assess optimal time for lung transplant referral (32). Royal Papworth Hospital (Cambridge, UK) are currently working with University of Cambridge (Cambridge, UK) and Microsoft Research (Redmond, WA, USA) to embed ML predictive analytics into “Project Breathe”, an ongoing international multicentre virtual monitoring study (33). Predictive analytics could be further enhanced with data from virtual monitoring topics previously discussed in this review such as symptoms, exercise, diet, and medication adherence.

Another application of AI is the use of Natural Language Processing (NLP) to analyse patient-generated data, such as symptom reports and treatment diaries. NLP algorithms can analyse these data and identify patterns and trends that may indicate changes in the patient's health status. This information can be used to provide personalised support and coaching to patients, and to alert healthcare providers to potential issues that may require intervention. In this space published research has looked at both processing unstructured data in the form of patient notes and online forums (34, 35).

AI could be used to develop predictive models to identify patients who are at high risk of pulmonary exacerbation, disease progression or non-adherence to their treatment plan. This information can be used to develop targeted interventions, such as personalised education and support, to improve adherence, and ultimately improve patient outcomes (36).

#### 3.1.2. New data generation and capturing

Much of the technology used by the different studies outlined in this review are now considered to be standardised. Namely PRO tools and Bluetooth® devices such as a portable spirometer, activity tracker, and thermometer. As such, future CF virtual monitoring should investigate means to capture certain medical data only obtainable within a hospital or lung function laboratory, or even new data itself. One such example could be the remote capturing of sweat chloride via portable-wearable sweat sensors (37). These sensors could have the potential to indicate if a patient is responding to modulator therapies and potentially assist in decision making regarding dose adjustments or provide more insight into the safety and efficacy of these therapies. Other novel data generation and capturing approaches could include the ability to analyse sputum samples at the patients home (point of care testing) (38), portable imaging devices (39), or portable listening apparatus such as a virtual stethoscope (40).

### 3.2. Barriers and considerations

#### 3.2.1. Data overload and interoperability

There has been an exponential growth in the volume of health data. IBM estimates that there are approximately 400 gigabytes of clinical data, six terabytes of omics data, and 1,100 terabytes of virtual monitoring recorded per patient over their lifetime (41). Consequently, when implementing virtual monitoring, it is important that only necessary data are recorded. In addition to this, consideration as to how virtual monitoring data will be integrated into hospital healthcare systems and/or patient health registries is required. This will prevent the siloing of data, potential duplication of data, and overloading the patient with virtual monitoring tasks.

#### 3.2.2. Digital divide

Virtual monitoring within CF creates opportunities to provide care to those who may otherwise be disadvantaged, for example geographical restriction, limited availability, potential communication barriers. However, it simultaneously introduces new potential for digital exclusion whereby if patient groups do not have access to the necessary hardware, software, and internet connection, they will be excluded. Furthermore, if the healthcare service lacks the required Information and Communication Technology (ICT) infrastructure and support, medical teams will be unable to support or fully utilise virtual monitoring techniques. Consequently, one of the biggest considerations for virtual monitoring in CF is the digital divide, and the need to provide medical teams with the correct support, and patients/families with the necessary technology (including a Wi-Fi connection) and education.

#### 3.2.3. Evaluation methods, outcome measures, and sustained Use

This review has highlighted some specific examples of where virtual monitoring could or has been used within CF. Many of these are small pilot studies evaluating usability and feasibility, while a smaller portion of studies include clinical parameters as part of their investigation. While both research methodologies contribute to the field there is need for the adoption of standardised evaluation and reporting methods. In addition to this, two studies commented on the inability to sustain virtual monitoring activity over time, and no studies discussed the costs associated with virtual monitoring. As such, there is need for real-world studies describing the experiences of all stakeholders with an emphasis on integration to sustain its use and all associated costs. This will avoid “pilotitis” and inform translational research and the integration of standardised virtual monitoring into CF care.

### 3.3. CF as an exemplar

Due to the multifaceted nature of CF, many virtual monitoring focuses outlined in previous sections can be translated to other medical conditions. Namely, remote recording of medical parameters, virtual clinics, or interventions for diet/exercise/self-management/treatment adherence. Similarly, many of the gaps in CF virtual monitoring are echoed in other medical contexts. In recent virtual monitoring reviews focusing on interstitial lung diseases (42), and cardiovascular disease (39) there are reoccurring gaps emerging such as digital exclusion caused by the digital divide, the understanding of virtual monitoring cost effectiveness, and strategies for its sustainability. These reviews also outline emerging virtual monitoring focuses not yet explored in CF such as remote/portable diagnostic imaging (39), and home sample testing (blood) (42). Therefore, the various virtual monitoring components in CF are anticipated to be informative to other chronic conditions, however there are novel virtual monitoring approaches that CF has yet to adopt and evaluate.

## 4. Conclusion

CF is a chronic condition with many associated co-morbidities. To support patients and families with their care and treatment, medical professionals and researchers have explored various virtual monitoring approaches. This review highlights some of the key studies investigating virtual monitoring in CF under different care components. Overall, virtual monitoring in all its forms has been shown to be acceptable, feasible, and usable with the potential to positively impact health outcomes. It also demonstrates many challenges within virtual monitoring, such as a lack of large clinical studies, standardised evaluation methods, the digital divide, and sustained use. While many traits of CF care and medicine are unique, the benefits and barriers to CF virtual monitoring are not, and can be translated to other medical focuses and chronic conditions.
